# Decrease in brain‐derived neurotrophic factor at plasma level but not in serum concentrations in suicide behavior: A systematic review and meta‐analysis

**DOI:** 10.1002/brb3.706

**Published:** 2017-04-19

**Authors:** Marisol Salas‐Magaña, Carlos A. Tovilla‐Zárate, Thelma B. González‐Castro, Isela E. Juárez‐Rojop, María L. López‐Narváez, José M. Rodríguez‐Pérez, Julián Ramírez Bello

**Affiliations:** ^1^ División Académica de Ciencias de la SaludUniversidad Juárez Autónoma de TabascoVillahermosaTabascoMéxico; ^2^ División Académica Multidisciplinaria de ComalcalcoUniversidad Juárez Autónoma de TabascoComalcalcoTabascoMéxico; ^3^ División Académica Multidisciplinaria de Jalpa de MéndezUniversidad Juárez Autónoma de TabascoJalpa de MéndezTabascoMéxico; ^4^ Hospital General de YajalónSecretaría de SaludYajalónChiapasMéxico; ^5^ Departamento de Biología MolecularInstituto Nacional de Cardiología Ignacio ChávezCiudad de MéxicoMéxico; ^6^ Unidad de InvestigaciónHospital Juárez de MéxicoSecretaría de SaludCiudad de MéxicoMéxico

**Keywords:** Brain‐derived neurotrophic factor, plasma, serum, suicide

## Abstract

**Introduction:**

Suicide is known as a major health concern worldwide. There is evidence for the role of brain‐derived neurotrophic factor (BDNF) in suicide behavior. Therefore, this factor has been proposed as a biomarker for suicide behavior. Clinical studies have measured BDNF concentrations at central and peripheral levels. As a consequence, the aim of this study was to assess BDNF levels in blood plasma and serum to see whether there is a difference in concentrations in patients with suicide behavior when compared to those in controls, using a meta‐analysis approach.

**Methods:**

We conducted a systematic review and meta‐analysis. The search strategy was performed using three databases: PubMed, EBSCO and ScienceDirect. The meta‐analysis included a total of nine case–control studies, six measured the BDNF level in serum and three in plasma in suicide behavior.

**Results:**

A decrease in BDNF levels in plasma was observed (*d *=* *−0.73, 95% CI −1.42 to −0.03 pg/ml). In the case of serum concentrations, no BDNF differences were encountered between cases and controls (*d* = 0.09, 95% CI −0.31 to 0.13 ng/ml, *p*(*Q*) = .92).

**Conclusions:**

According to the results found in the present meta‐analysis, the plasma BDNF level could be suggest as a potential biomarker in suicide behavior. However, since the number of studies included in the analysis is limited, a larger number is necessary to determine conclusively the role of BDNF as a biomarker in suicide behavior.

## 1 Introduction

The suicide behavior comprises the ideation of the suicide, the intent and the suicide (Turecki & Brent, 2016). Suicide represents today a major public health problem. According to the WHO organization, over 800,000 people die by this cause every year in the world (WHO, 2014). Even though various risk factors have been identified such as vulnerable age groups, psychiatric disorders, drug and alcohol use, social and economic issues among them (Hegerl, 2016; Hernandez‐Alvarado et al., 2016; Johnson, Carver, & Tharp, 2016; Ventriglio et al., 2016), there are some exceptions that cannot be explained by these causes (Costanza, Baertschi, Weber, & Canuto, 2015). In the etiology of suicide behavior, one major issue that still offers a limited understanding is the neurobiological aspect (Lindqvist et al., 2011). Biomarkers, substances used as indicators of a biological state, represent a useful tool in the diagnosis and prediction of suicide behavior (Le‐Niculescu et al., 2013; Strimbu & Tavel, 2010). One of the main biomarkers proposed in association studies with suicide attempted and suicide is a neurotrophin named brain‐derived neurotrophic factor (BDNF; Grah et al., 2014; Priya, Rajappa, Kattimani, Mohanraj, & Revathy, 2016).

In the human body, the concentration of BDNF is higher in cerebral tissue than in the blood stream (Hashimoto, 2010). Of the BDNF present in the circulation, more than a half is originated from brain structures (Rasmussen et al., 2009). Recently, BDNF has been implicated as a possible biomarker for psychiatric diseases such as schizophrenia, bipolar disorder and major depression, given that BDNF levels are lower in these patients than in healthy subjects (Castren & Kojima, 2017; Eisen et al., 2015; Grah et al., 2014; Hashimoto, 2010; Kim et al., 2007; Lee & Kim, 2009; Priya et al., 2016). Dwivedi et al. (2003) reported a decrease in BDNF levels in patients that completed the suicide. Since then, the interest in BDNF levels as a biomarker in suicide behavior has been on the rise. Furthermore, in suicide attempt, a decrease between serum BDNF levels and patients with this behavior has been observed (Eisen et al., 2016). At the moment, one meta‐analysis has been conducted to study the association between BDNF levels and suicide behavior (Eisen et al., 2015); this analysis only included three articles. The findings of this meta‐analysis showed no association between serum BDNF levels and suicide attempt.

Given the hypothesis that the BDNF could be used as a biomarker, we decide to perform a meta‐analysis of BDNF levels measured in two types of samples: plasma, serum for suicide attempt and hippocampus in suicide. To probe this hypothesis, we performed an exploratory study through a meta‐analysis to evaluate the differences between suicide attempters and controls in both in serum and plasma BDNF levels to determine whether the BDNF level could be used as biomarker among individuals with suicide behavior.

## 2 Methods

This study was designed as a systematic review and meta‐analysis to evaluate the possible participation of the BDNF level in plasma, serum for suicide attempt and hippocampus tissue for suicide as biomarker in the etiology of suicide. Both methodological approaches were performed according to the Preferred Reporting Items for Systematic Reviews and Meta‐Analyses (PRISMA) guidelines.

The protocol registration in PROSPERO is CRD42016048612.

### 2.1 Search strategies

The electronic search was carried out in three different databases, PubMed, EBSCO, and ScienceDirect. The literature review covered the period between June and July 2016 and the search was conducted using the queries required to maximize the sensitivity of the literature search, viz.: BDNF plasma levels and suicide (PubMed: 15; EBSCO: 9; ScienceDirect: 880), BDNF serum levels and suicide (PubMed: 16; EBSCO: 9: ScienceDirect: 898), BDNF brain and suicide (PubMed: 125; EBSCO: 82; ScienceDirect: 1,892), BDNF and suicide (PubMed: 133; EBSCO: 100; ScienceDirect: 1,979), BDNF and suicidal behavior (PubMed: 83; EBSCO: 63; ScienceDirect: 785).

### 2.2 Inclusion and exclusion criteria

The inclusion criteria were the following: (1) That the study had to be published in a peer‐reviewed journal; (2) That the reports were association cases‐control studies type; (3) Reported BDNF concentrations of plasma or serum in suicide attempters; (4) Reported BDNF hippocampal measures in patients with suicide. (5) Studies published in English.

As exclusion criteria: (1) studies lacking the standard derivation of the mean BDNF levels were excluded from the analysis (Grah et al., 2014; Mansur et al., 2016; Priya et al., 2016). We contacted the corresponding authors to obtain the standard deviation and missing data. (2) Studies that provided analysis in different tissue to hippocampus (Martinez, Garakani, Yehuda, & Gorman, 2012).

### 2.3 Data extraction

To determine the descriptive characteristic of the studies, we extracted the following data for the systematic review: authors, year of publication, region, ethnic origin, sample size, number of cases and controls, gender, age, levels of BDNF in serum, plasma and brain tissue (hippocampus), and psychiatric diagnosis. Two researchers (Salas‐Magaña and González‐Castro) screened independently each article by title, abstract and full text to determine inclusion and selection of the studies. A consensus of the information was reached.

### 2.4 Measure of quality

The studies were assessed using the Grading of Recommendations Assessment, Development and Evaluation Scale system (http://www.gradeworkinggroup.org) to evaluate quality. In addition of the quality assessment, the Newcastle‐Ottawa Scale was also applied to the selected studies (http://www.ohri.ca/programs/clinical_epidemiology/oxford.asp). We made an assessment to distinguish high quality studies from the rest by using a mark of six score points or more.

### 2.5 Statistical analysis

We grouped studies according to the type of sample ‐plasma, serum for suicide attempt and hippocampus for suicide‐ and made the comparison for each case. If multiple case‐groups existed within a certain category (e.g., depressed with or without suicide attempt), a collection was made for each of them. For the meta‐analysis, only attempted suicide and completed suicide groups were included, however, the mean age and standard deviation (*SD*) in some cases were taken from the total number of cases. When studies assaying BDNF from brain tissue consisted of multiple analytical sections (e.g., PFC, entorhinal cortex, basolateral nucleus of amygdala [BLA], CFS), only the hippocampus data was included in the analysis.

To determine whether BDNF could be used as a biomarker, the outcomes of the meta‐analyses are expressed as standardized mean difference (*d*) and 95% CI (Perroud et al., 2009). The standardized mean difference was calculated from the mean and *SD* of BDNF levels for cases and control groups. All analyses were conducted in EPIDAT 3.1 software (http://www.sergas.es/Saude-publica/Epidat-3-1-descargar-Epidat-3-1-(espanol)?print=1). To assess heterogeneity among studies we used the Dersimonian and Laird's *Q* test and inconsistence index (*I* ^2^). Likewise, publication bias was analyzed by the Egger's test. A series of analyses was performed according to the type of sample (serum, plasma and brain tissue). The aim of this study was to determine whether BDFN levels were lower in suicide cases (suicide ideation, suicide attempt and completed suicide) than in healthy controls. A sensitivity analysis was performed to assess the influence of each study in the global estimation. Finally, publication bias was explored using the Egger's test and shown as funnel plots using the EPIDAT 3.1 Software.

## 3 Results

### 3.1 Study information

**Figure 1:**
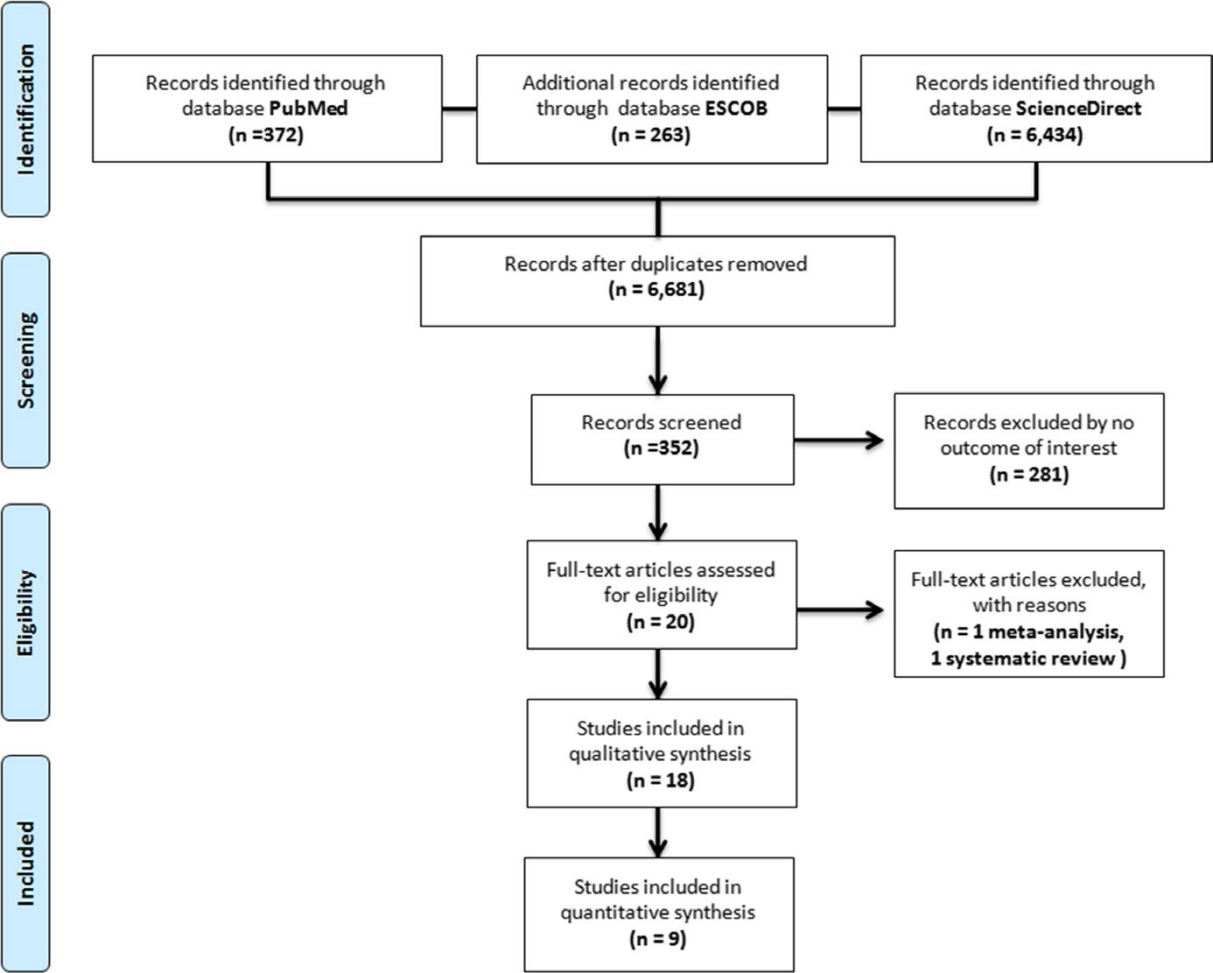
Flow‐diagram illustrating the search and inclusion/exclusion criteria used for the meta‐analysis and systematic review according to the Preferred Reporting Items for Systematic Reviews and Meta‐Analyses statement criteria

In our screening phase, our search strategy retrieved 7,069 potentially relevant studies. Next, we removed 388 duplicated articles, so 6,681 articles were assessed. Figure 1 summarizes the stages of the meta‐analysis in the PRISMA flow chart. The combined data of 16 studies (before the exclusion criteria were applied) are presented in Tables 1 and 2. In the end only nine studies fulfilled our inclusion criteria; these studies were performed in Korea (Kim et al., 2007; Lee & Kim, 2009; Lee, Kim, Park, & Kim, 2007; Park, Lee, Um, & Kim, 2014); Taiwan (Huang & Lee, 2006); Turkey (Deveci, Aydemir, Taskin, Taneli, & Esen‐Danaci, 2007); China (Liang, Zhang, Hong‐Ya, & Lv, 2012); Canada (Eisen et al., 2016), and Brazil (Pinheiro et al., 2012). As a result, in this meta‐analysis we included 246 cases and 601 controls overall, which were distributed in 80 cases and 145 controls for the meta‐analysis of plasma samples; 166 cases and 456 control for the meta‐analysis of serum samples. The meta‐analysis of hippocampal samples was not performed because the measuring units did not coincide in these studies (Dwivedi et al., 2003; Karege, Vaudan, Schwald, Perroud, & La Harpe, 2005; Table 1).

**Table 1:** Descriptive characteristics of the studies included in the systematic review and meta‐analysis in plasma and serum

Reference	Location	Diagnosis	Sample	Units	Cases			Controls		
					*n*	Mean	*SD*	*n*	Mean	*SD*
Lee et al. (2007)	Korea	MDD	Plasma	pg/ml	28	386.61	362.39	95	819.20	347.05
Kim et al. (2007)	Korea	MDD	Plasma	pg/ml	32	430.5	397.00	30	889.40	611.30
Lee and Kim (2009)	Korea	MDD	Plasma	pg/ml	20	713.04	236.56	20	709.05	172.12
Huang and Lee (2006)	Taiwan	SCZ	Serum	ng/ml	11	14.60	7.02	96	14.17	6.86
Deveci et al. (2007)	Turkey	AD	Serum	ng/ml	10	21.20	12.24	26	31.4	8.80
Liang et al. (2012)	China	MDD	Serum	ng/ml	31	57.30	9.20	30	113.8	44.4
Park et al. (2014)	Korea	MDD	Serum	ng/ml	18	21.93	24.71	33	24.71	7.7
Pinheiro et al. (2012)	Brazil	PPAD	Serum	ng/ml	12	2.11	1.42	178	2.37	1.26
Eisen et al. (2016)	Canada	MAD	Serum	ng/ml	84	24.21	7.19	93	24.77	7.01

MDD, major depression disorder; SZC, schizophrenia; AD, adjustment disorder; PPAD, postpartum affective disorder; MAD, mood and anxiety disorder.

**Table 2:** Descriptive characteristics of the studies no included in the meta‐analysis

Reference	Location	Diagnosis	Sample	Units	Cases				Controls			
					*n*	Subgroups	mean	*SD*	*n*	Subgroups	Mean	*SD*
Karege et al. (2005)	Switzerland	MDD, BD	Hippocampus	ng/g	22	Drug‐free MDD	17.7	2.9	8	Drug‐free	24.5	3.6
						Drug‐free others	16.8	3.1				
						Drug‐treated MDD	23.3	2.2				
Banerjee et al. (2013)	India	MDD, BD, AD, SCZ, FD, MD	Hippocampus	pg/ml	21		19.5	—	19		44	—
Dwivedi et al. (2003)	EE.UU.	MDD, AD, BD, SCZ	Hippocampus	Optical density	27	Suicide/MDD	1.04	0.20	21		1.71	0.44
						Suicide/OPD	1.03	0.22				
Hayley et al. (2015)	Hungary	MDD	Hippocampus	Optical density	9	Females‐Hippocampus	0.65	—	10	Females‐Hippocampus	0.55	—
					10	Males‐Hippocampus	0.40		9	Males‐Hippocampus	0.75	
Mansur et al. (20160	Brazil	BD	Plasma	pg/ml	57			—	26			—
Grah et al. (2014)	Croatia	AD, MDD	Serum	ng/ml	96	(26) RDD	11.8	—	106	(60) C	13.40	—
						(33) PD	10.7			(25) RDD	12.80	
						(37) AD	12.6			(26) PD	15.70	
										(25) AD	15.40	
Martinez et al. (2012)	EE.UU.	MDD	CFS	pg/ml	18			—	25			—
Hayley et al. (2015)	Hungary	MDD	PFC, hippocampus	Optical density	9	Females		—	10	Females		—
						PFC	0.75			PFC	1.40	
						Hippocampus	0.65			Hippocampus	0.55	
					10	Males			9	Males		
						PFC	0.50			PFC	0.40	
						Hippocampus	0.40			Hippocampus	0.75	

MDD, major depression disorder; BD, bipolar disorder; AD, adjustment disorder; SZC, schizophrenia; FD, familiar disharmony; MD, marital disharmony; OPD, other psychiatric disorder; RDD, recurrent depressive disorder; MAD, mood and anxiety disorder; PD, psychiatric disorder; PPAD, postpartum affective disorder; PFC, prefrontal cortex; C, control.

### 3.2 Meta‐analysis of BDNF levels in plasma

**Figure 2:**
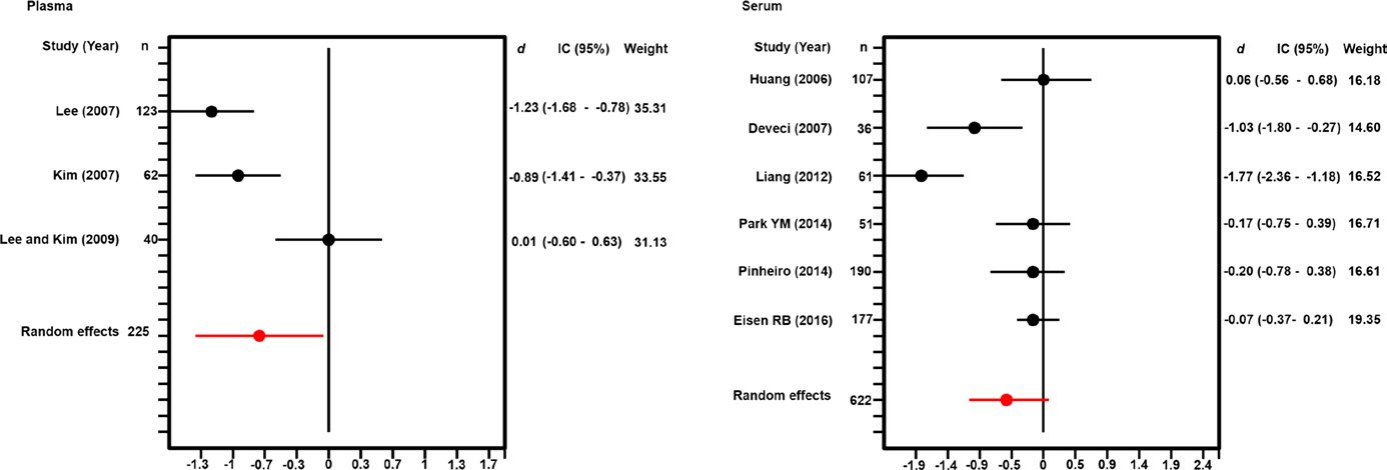
Forest plots of the meta‐analysis of brain‐derived neurotrophic factor levels measured in blood plasma and serum

First, we evaluated BDNF levels in plasma and found that values were lower in cases than in controls. The case–control studies analyzing BDNF levels in plasma were 3 (Kim et al., 2007; Lee & Kim, 2009; Lee et al., 2007). The results of the standardized mean difference showed lower BDNF levels in the cases with suicide attempt than in healthy controls (*d *=* *−0.73, 95% CI −1.42 to −0.03 pg/ml, *I* ^2^ = 80.74, *p*(*Q*) < .01 (Figure 1a). Our results suggest that BDNF levels are decreased in plasma in patients with suicide behavior when compared to control groups. The Forest plots of the meta‐analysis of BDNF and levels in plasma is showed in the Figure 2.

### 3.3 Meta‐analysis of BDNF levels in serum

Subsequently, we conducted a meta‐analysis to assess whether BDNF levels in serum were lower in cases than in controls. In this analysis the sum of samples consisted of six studies that evaluated the association between BDNF levels in serum for cases with suicide attempt and controls (Deveci et al., 2007; Eisen et al., 2015; Huang & Lee, 2006; Liang et al., 2012; Park et al., 2014; Pinheiro et al., 2012). The analysis did not show statistical difference in serum BDNF levels between cases and control groups (*d* = −0.51, 95% CI −1.06 to 0.33 ng/ml, *I* ^2^ = 83.79, *p*(*Q*) < .001 (Figure 1b). The Forest plots of the meta‐analysis of BDNF and levels in serum is showed in Figure 2.

### 3.4 BDNF levels in brain tissue

The type of sample of the studies varied. The studies found used different parts of the postmortem brain of suicide victims. We first group the studies according to their similarity in study sample. While some used hippocampus (Banerjee, Ghosh, Ghosh, Bhattacharyya, & Mondal, 2013; Dwivedi et al., 2003; Hayley et al., 2015; Karege et al., 2005), others utilized BLA and central nucleus of amygdala (Maheu, Davoli, Turecki, & Mechawar, 2013) and also the *cerebrospinal fluid* (Martinez et al., 2012).

With respect to the studies with BDNF hippocampal levels the findings the followings. In the study performed in Switzerland by Karege et al. (2005), reported a BDNF decrease in suicide victims, regardless of diagnosis, also observed in an American population (Dwivedi et al., 2003). This decrease was observed also in an Indian population (Banerjee et al., 2013), even when the sample present several psychiatric diagnoses such as major depress disorder, bipolar disorder and adjustment disorder. In the last study performed in Hungary (Hayley et al., 2015), the results varied according the gender. The male suicides present significant reductions of BDFN levels, but not female suicides.

## 4 Discussion

In this study, we performed a systematic review and meta‐analysis to evaluate whether BDNF levels (serum, plasma for suicide attempt and BDNF level in hippocampus for suicide) were decreased in patients with suicide behavior. Our purpose was to determine whether BDNF could be used as a biomarker among individuals with suicide behavior. In our meta‐analysis of peripheral samples, we found that in plasma but not in serum BDNF levels were decreased in patients with suicide attempt. To the best of our knowledge, this study is the first meta‐analysis showing a decrease in BDNF levels in plasma for this type of patients. Our results support the hypothesis that some neurotrophins are implicated in neuropsychiatric disorders and that BDNF could be implicated in suicide behavior (Eisen et al., 2016).

A possible explanation for low peripheral BDNF levels in patients with suicide attempt could be that severe stress consequent with attempted suicide may markedly decrease BDNF levels after the attempt. Another probable cause for low peripheral BDNF levels in patients with suicide attempt could be a down‐regulation of BDNF expression derived from a decrease in serotonin function in central level (Ambrus, Lindqvist, Traskman‐Bendz, & Westrin, 2016). However, the evidence should be taken with caution, since up to now only three studies in the literature have examined BDNF levels in plasma (Eisen et al., 2016; Kim et al., 2007; Lee & Kim, 2009; Lee et al., 2007) and the assayed sample was small (80 cases and 145 controls).

Second, a different scenario was encountered when the meta‐analysis was addressed to assess BDNF levels in serum. In this case, no significant differences in BDNF concentrations were observed between patients with suicide attempt and controls. Our results are in agreement with a previous meta‐analysis (Eisen et al., 2015). We could not find a significant association between BDNF in serum and suicide attempt. In this study, we included 168 cases and 326 controls more and we included six publications, three more than those reported in the previous meta‐analysis. The two meta‐analyses showed a no association, between BDNF levels in serum and suicide attempt.

The apparent contradiction in peripheral BDNF levels (decrease level of BDNF in plasma but not in serum) could be explained due the different mechanism that both, serum and plasma have (Fernandes, Molendijk, et al., 2015; Fujimura et al., 2002). BDNF is released into the internal jugular vein, suggesting that the brain delivers it to the circulation. Almost three‐quarters of the BDNF present in circulation are originated from brain structures, suggesting that brain is the main contributor to the circulating BDNF (Krabbe et al., 2007; Rasmussen et al., 2009). It is known to be stored in human platelets and to circulate in plasma (Lommatzsch et al., 2005). The literature shows that BDNF in plasma is released upon agonist stimulation (Fujimura et al., 2002). Based on the above, the alterations in plasma BDNF levels could reflect more specific changes at central nervous system level than alterations in serum BDNF levels. This situation has been previously demonstrated in schizophrenia and bipolar disorders, which are at the same time, part of the risk factors in suicide and suicide behavior (Fernandes, Molendijk, et al., 2015; Fernandes, Steiner, et al., 2015; Fujimura et al., 2002). Then, this assumption could also be true for this major public health problem. However, this asseveration could gain more support from the evidence.

Finally, in this study, we evaluate the BDNF levels in a central way through the meta‐analysis. The brain tissue used in them was the hippocampus (Banerjee et al., 2013; Dwivedi et al., 2003; Hayley et al., 2015; Karege et al., 2005). But at the moment, the measuring units do not coincide in the different studies, so it is not possible to perform a meta‐analysis of hippocampus BDNF levels in suicide behavior (Fujimura et al., 2002).

The present study has some limitations. First, although our meta‐analysis involves assessments of BDNF levels in plasma and serum of patients with suicide behavior and includes more studies and subjects than the past meta‐analysis, the number of studies is still small. The number the studies is even smaller than other studies analyzing BDNF levels in other diseases such as bipolar disorder (Fernandes, Molendijk, et al., 2015) or schizophrenia. Second, the comorbidity in the patients diagnosis, such as schizophrenia, major depress disorder, bipolarity and adjustment disorder, to name some of them, could be considered in the interpretation of the results. In consequence, more studies are necessary to analyze comorbidity of specific groups. Third, the time between the date of suicide attempt and the tissue sampling is not known. We could not analyze the relation between the time of the suicide attempt and BDNF concentrations in plasma or serum. Similarly, it is also necessary to analyze whether the number of suicide attempts is associated with a higher reduction in BDNF levels. At the last, as a methodological observation all the studies performed the measures of plasma and serum by ELISA.

Finally, we believe that altered BDNF levels in plasma observed in the analysis influence remarkably in the suicide attempt and suicide. Therefore, it is important to continue the searching of how this altered levels may be influenced at a central level (Serra‐Millàs, 2016). Also, it would be necessary evaluate certain factors, like the environment, the family experience and the precedents in childhood and the three stages of suicide behavior.

In summary, the present meta‐analysis suggests potential differences between BDNF levels in plasma and serum and suggests that BDNF could be used as a potential biomarker in suicide attempt. However, as the number of studies included in the present meta‐analysis is limited, future studies are necessary to measure BDNF levels in plasma, so that these results can be confirmed in larger samples and in different populations.

## Conflict of Interest

The authors declare not to have any competing interests.

## References

[brb3706-bib-0001] Ambrus, L. , Lindqvist, D. , Traskman‐Bendz, L. , & Westrin, A. (2016). Hypothalamic‐pituitary‐adrenal axis hyperactivity is associated with decreased brain‐derived neurotrophic factor in female suicide attempters. Nordic Journal of Psychiatry, 70(8), 575–581. 27216156 10.1080/08039488.2016.1184310

[brb3706-bib-0002] Banerjee, R. , Ghosh, A. K. , Ghosh, B. , Bhattacharyya, S. , & Mondal, A. C. (2013). Decreased mRNA and protein expression of BDNF, NGF, and their receptors in the hippocampus from suicide: An analysis in human postmortem brain. Clinical Medicine Insights: Pathology, 6, 1–11. doi:10.4137/CMPath.S12530 24031163 10.4137/CPath.S12530 PMC3767649

[brb3706-bib-0003] Castren, E. , & Kojima, M. (2017). Brain‐derived neurotrophic factor in mood disorders and antidepressant treatments. Neurobiology of Diseases, 97(Part B), 119–126. doi:10.1016/j.nbd.2016.07.010 10.1016/j.nbd.2016.07.010 27425886

[brb3706-bib-0004] Costanza, A. , Baertschi, M. , Weber, K. , & Canuto, A. (2015). Neurological diseases and suicide: From neurobiology to hopelessness. Revue Médicale Suisse, 11(461), 402–405. 25895218

[brb3706-bib-0005] Deveci, A. , Aydemir, O. , Taskin, O. , Taneli, F. , & Esen‐Danaci, A. (2007). Serum BDNF levels in suicide attempters related to psychosocial stressors: A comparative study with depression. Neuropsychobiology, 56(2–3), 93–97. doi:10.1159/000111539 18037819 10.1159/000111539

[brb3706-bib-0006] Dwivedi, Y. , Rizavi, H. S. , Conley, R. R. , Roberts, R. C. , Tamminga, C. A. , & Pandey, G. N. (2003). Altered gene expression of brain‐derived neurotrophic factor and receptor tyrosine kinase B in postmortem brain of suicide subjects. Archives of General Psychiatry, 60(8), 804–815. doi:10.1001/archpsyc.60.8.804 12912764 10.1001/archpsyc.60.8.804

[brb3706-bib-0007] Eisen, R. B. , Perera, S. , Banfield, L. , Anglin, R. , Minuzzi, L. , & Samaan, Z. (2015). Association between BDNF levels and suicidal behaviour: A systematic review and meta‐analysis. Systematic Reviews, 4, 187. doi:10.1186/s13643‐015‐0179‐z 26718989 10.1186/s13643-015-0179-z PMC4697315

[brb3706-bib-0008] Eisen, R. B. , Perera, S. , Bawor, M. , Dennis, B. B. , El‐Sheikh, W. , DeJesus, J. , … Samaan, Z. (2016). Exploring the association between serum BDNF and attempted suicide. Scientific Reports, 6, 25229. doi:10.1038/srep25229 27121496 10.1038/srep25229 PMC4848497

[brb3706-bib-0009] Fernandes, B. S. , Molendijk, M. L. , Kohler, C. A. , Soares, J. C. , Leite, C. M. , Machado‐Vieira, R. , … Carvalho, A. F. (2015). Peripheral brain‐derived neurotrophic factor (BDNF) as a biomarker in bipolar disorder: A meta‐analysis of 52 studies. BMC Medicine, 13, 289. doi:10.1186/s12916‐015‐0529‐7 26621529 10.1186/s12916-015-0529-7 PMC4666054

[brb3706-bib-0010] Fernandes, B. S. , Steiner, J. , Berk, M. , Molendijk, M. L. , Gonzalez‐Pinto, A. , Turck, C. W. , … Goncalves, C. A. (2015). Peripheral brain‐derived neurotrophic factor in schizophrenia and the role of antipsychotics: Meta‐analysis and implications. Molecular Psychiatry, 20(9), 1108–1119. doi:10.1038/mp.2014.117 25266124 10.1038/mp.2014.117

[brb3706-bib-0011] Fujimura, H. , Altar, C. A. , Chen, R. , Nakamura, T. , Nakahashi, T. , Kambayashi, J. , … Tandon, N. N. (2002). Brain‐derived neurotrophic factor is stored in human platelets and released by agonist stimulation. Thrombosis and Haemostasis, 87(4), 728–734. 12008958

[brb3706-bib-0012] Grah, M. , Mihanovic, M. , Ruljancic, N. , Restek‐Petrovic, B. , Molnar, S. , & Jelavic, S. (2014). Brain‐derived neurotrophic factor as a suicide factor in mental disorders. Acta Neuropsychiatrica, 26(6), 356–363. doi:10.1017/neu.2014.27 25308403 10.1017/neu.2014.27

[brb3706-bib-0013] Hashimoto, K. (2010). Brain‐derived neurotrophic factor as a biomarker for mood disorders: An historical overview and future directions. Psychiatry and Clinical Neurosciences, 64(4), 341–357. doi:10.1111/j.1440‐1819.2010.02113.x 20653908 10.1111/j.1440-1819.2010.02113.x

[brb3706-bib-0014] Hayley, S. , Du, L. , Litteljohn, D. , Palkovits, M. , Faludi, G. , Merali, Z. , … Anisman, H. (2015). Gender and brain regions specific differences in brain derived neurotrophic factor protein levels of depressed individuals who died through suicide. Neuroscience Letters, 600, 12–16. doi:10.1016/j.neulet.2015.05.052 26033186 10.1016/j.neulet.2015.05.052

[brb3706-bib-0015] Hegerl, U. (2016). Prevention of suicidal behavior. Dialogues in Clinical Neuroscience, 18(2), 183–190. 27489458 10.31887/DCNS.2016.18.2/uhegerl PMC4969705

[brb3706-bib-0016] Hernandez‐Alvarado, M. M. , Gonzalez‐Castro, T. B. , Tovilla‐Zarate, C. A. , Fresan, A. , Juarez‐Rojop, I. E. , Lopez‐Narvaez, M. L. , … Genis‐Mendoza, A. (2016). Increase in suicide rates by hanging in the population of Tabasco, Mexico between 2003 and 2012. International journal of environmental research and public health, 13(6), doi:10.3390/ijerph13060552 10.3390/ijerph13060552 PMC4924009 27258292

[brb3706-bib-0017] Huang, T. L. , & Lee, C. T. (2006). Associations between serum brain‐derived neurotrophic factor levels and clinical phenotypes in schizophrenia patients. Journal of Psychiatric Research, 40(7), 664–668. doi:10.1016/j.jpsychires.2005.11.004 16386272 10.1016/j.jpsychires.2005.11.004

[brb3706-bib-0018] Johnson, S. L. , Carver, C. S. , & Tharp, J. A. (2016). Suicidality in bipolar disorder: The role of emotion‐triggered impulsivity. Suicide and Lifethreatening Behavior. doi:10.1111/sltb.12274 10.1111/sltb.12274 PMC5788807 27406282

[brb3706-bib-0019] Karege, F. , Vaudan, G. , Schwald, M. , Perroud, N. , & La Harpe, R. (2005). Neurotrophin levels in postmortem brains of suicide victims and the effects of antemortem diagnosis and psychotropic drugs. Molecular Brain Research, 136(1–2), 29–37. doi:10.1016/j.molbrainres.2004.12.020 15893584 10.1016/j.molbrainres.2004.12.020

[brb3706-bib-0020] Kim, Y. K. , Lee, H. P. , Won, S. D. , Park, E. Y. , Lee, H. Y. , Lee, B. H. , … Choi, S. H. (2007). Low plasma BDNF is associated with suicidal behavior in major depression. Progress in Neuro‐Psychopharmacology and Biological Psychiatry, 31(1), 78–85. doi:10.1016/j.pnpbp.2006.06.024 16904252 10.1016/j.pnpbp.2006.06.024

[brb3706-bib-0021] Krabbe, K. S. , Nielsen, A. R. , Krogh‐Madsen, R. , Plomgaard, P. , Rasmussen, P. , Erikstrup, C. , … Pedersen, B. K. (2007). Brain‐derived neurotrophic factor (BDNF) and type 2 diabetes. Diabetologia, 50(2), 431–438. doi:10.1007/s00125‐006‐0537‐4 17151862 10.1007/s00125-006-0537-4

[brb3706-bib-0022] Lee, B. H. , & Kim, Y. K. (2009). Reduced platelet BDNF level in patients with major depression. Progress in Neuro‐Psychopharmacology and Biological Psychiatry, 33(5), 849–853. doi:10.1016/j.pnpbp.2009.04.002 19371767 10.1016/j.pnpbp.2009.04.002

[brb3706-bib-0023] Lee, B. H. , Kim, H. , Park, S. H. , & Kim, Y. K. (2007). Decreased plasma BDNF level in depressive patients. Journal of Affective Disorders, 101(1–3), 239–244. doi:10.1016/j.jad.2006.11.005 17173978 10.1016/j.jad.2006.11.005

[brb3706-bib-0024] Le‐Niculescu, H. , Levey, D. , Ayalew, M. , Palmer, L. , Gavrin, L. , Jain, N. , … Radel, M. (2013). Discovery and validation of blood biomarkers for suicidality. Molecular Psychiatry, 18(12), 1249–1264. 23958961 10.1038/mp.2013.95 PMC3835939

[brb3706-bib-0025] Liang, W. , Zhang, H.‐M. , Zhang, H.‐Y. , & Lv, L.‐X. (2012). Association of brain‐derived neurotrophic factor protein in peripheral blood and gene expression to suicidal behavior in patients with depression. Chinese Mental Health Journal, 26(10), 726–730.

[brb3706-bib-0026] Lindqvist, D. , Janelidze, S. , Erhardt, S. , Träskman‐Bendz, L. , Engström, G. , & Brundin, L. (2011). CSF biomarkers in suicide attempters—A principal component analysis. Acta Psychiatrica Scandinavica, 124(1), 52–61. doi:10.1111/j.1600‐0447.2010.01655.x 21198458 10.1111/j.1600-0447.2010.01655.x

[brb3706-bib-0027] Lommatzsch, M. , Zingler, D. , Schuhbaeck, K. , Schloetcke, K. , Zingler, C. , Schuff‐Werner, P. , & Virchow, J. C. (2005). The impact of age, weight and gender on BDNF levels in human platelets and plasma. Neurobiology of Aging, 26(1), 115–123. doi:10.1016/j.neurobiolaging.2004.03.002 15585351 10.1016/j.neurobiolaging.2004.03.002

[brb3706-bib-0028] Maheu, M. E. , Davoli, M. A. , Turecki, G. , & Mechawar, N. (2013). Amygdalar expression of proteins associated with neuroplasticity in major depression and suicide. Journal of Psychiatric Research, 47(3), 384–390. doi:10.1016/j.jpsychires.2012.11.013 23260340 10.1016/j.jpsychires.2012.11.013

[brb3706-bib-0029] Mansur, R. B. , Santos, C. M. , Rizzo, L. B. , Asevedo, E. , Cunha, G. R. , Noto, M. N. , … Brietzke, E. (2016). Brain‐derived neurotrophic factor, impaired glucose metabolism, and bipolar disorder course. Bipolar Disorders, 18(4), 373–378. doi:10.1111/bdi.12399 27324989 10.1111/bdi.12399

[brb3706-bib-0030] Martinez, J. M. , Garakani, A. , Yehuda, R. , & Gorman, J. M. (2012). Proinflammatory and “resiliency” proteins in the CSF of patients with major depression. Depression and anxiety, 29(1), 32–38. doi:10.1002/da.20876 21898706 10.1002/da.20876

[brb3706-bib-0031] Park, Y. M. , Lee, B. H. , Um, T. H. , & Kim, S. (2014). Serum BDNF levels in relation to illness severity, suicide attempts, and central serotonin activity in patients with major depressive disorder: A pilot study. PLoS One, 9(3), e91061. doi:10.1371/journal.pone.0091061 24663244 10.1371/journal.pone.0091061 PMC3963843

[brb3706-bib-0032] Perroud, N. , Aitchison, K. J. , Uher, R. , Smith, R. , Huezo‐Diaz, P. , Marusic, A. , … Craig, I. (2009). Genetic predictors of increase in suicidal ideation during antidepressant treatment in the GENDEP project. Neuropsychopharmacology, 34(12), 2517–2528. doi:10.1038/npp.2009.81 19641488 10.1038/npp.2009.81

[brb3706-bib-0033] Pinheiro, R. T. , Pinheiro, K. A. , da Cunha Coelho, F. M. , de Avila Quevedo, L. , Gazal, M. , da Silva, R. A. , … Oses, J. P. (2012). Brain‐derived neurotrophic factor levels in women with postpartum affective disorder and suicidality. Neurochemical Research, 37(10), 2229–2234. doi:10.1007/s11064‐012‐0851‐9 22851352 10.1007/s11064-012-0851-9

[brb3706-bib-0034] Priya, P. K. , Rajappa, M. , Kattimani, S. , Mohanraj, P. S. , & Revathy, G. (2016). Association of neurotrophins, inflammation and stress with suicide risk in young adults. Clinica Chimica Acta, 457, 41–45. doi:10.1016/j.cca.2016.03.019 10.1016/j.cca.2016.03.019 27034054

[brb3706-bib-0035] Rasmussen, P. , Brassard, P. , Adser, H. , Pedersen, M. V. , Leick, L. , Hart, E. , … Pilegaard, H. (2009). Evidence for a release of brain‐derived neurotrophic factor from the brain during exercise. Experimental Physiology, 94(10), 1062–1069. doi:10.1113/expphysiol.2009.048512 19666694 10.1113/expphysiol.2009.048512

[brb3706-bib-0036] Serra‐Millàs, M. (2016). Are the changes in the peripheral brain‐derived neurotrophic factor levels due to platelet activation? World Journal of Psychiatry, 6(1), 84–101. doi:10.5498/wjp.v6.i1.84 27014600 10.5498/wjp.v6.i1.84 PMC4804271

[brb3706-bib-0037] Strimbu, K. , & Tavel, J. A. (2010). What are biomarkers? Current opinion in HIV and AIDS, 5(6), 463–466. doi:10.1097/COH.0b013e32833ed177 20978388 10.1097/COH.0b013e32833ed177 PMC3078627

[brb3706-bib-0038] Turecki, G. , & Brent, D. A. (2016). Suicide and suicidal behaviour. Lancet, 387(10024), 1227–1239. doi:10.1016/s0140‐6736(15)00234‐2 26385066 10.1016/S0140-6736(15)00234-2 PMC5319859

[brb3706-bib-0039] Ventriglio, A. , Gentile, A. , Bonfitto, I. , Stella, E. , Mari, M. , Steardo, L. , & Bellomo, A. (2016). Suicide in the early stage of schizophrenia. Frontiers in Psychiatry, 7, 116. doi:10.3389/fpsyt.2016.00116 27445872 10.3389/fpsyt.2016.00116 PMC4921745

[brb3706-bib-0040] WHO (2014). Preventing suicide: A global imperative. Switzerland: Author.

